# Individually tailored exercise in patients with postural orthostatic tachycardia syndrome related to post-COVID-19 condition – a feasibility study

**DOI:** 10.1038/s41598-024-71055-5

**Published:** 2024-08-28

**Authors:** Annie Svensson, Anna Svensson-Raskh, Linda Holmström, Carl Hallberg, Lucian Bezuidenhout, David Moulaee Conradsson, Marcus Ståhlberg, Judith Bruchfeld, Artur Fedorowski, Malin Nygren-Bonnier

**Affiliations:** 1 Division of Physiotherapy, Department of Neurobiology, Care Sciences and Society, Karolinska Institutet, Stockholm, Sweden; 2 Medical Unit Allied Health Professionals, Women’s Health and Allied Health Professionals Theme, Karolinska University Hospital, Stockholm, Sweden; 3 Department of Clinical Neuroscience, Karolinska Institutet, Stockholm, Sweden; 4 Department of Women’s and Children’s Health, Karolinska Institutet, Stockholm, Sweden; 5 Division of Physiotherapy, Department of Health and Rehabilitation Sciences, Stellenbosch University, Cape Town, South Africa; 6 Department of Medicine, Karolinska Institutet, Solna, Sweden; 7 Department of Cardiology, Karolinska University Hospital, Stockholm, Sweden; 8 Department of Infectious Diseases, Karolinska University Hospital, Stockholm, Sweden; 9 Division of Infectious Diseases, Department of Medicine Solna, Karolinska Institutet, Stockholm, Sweden

**Keywords:** Quality of life, Rehabilitation

## Abstract

Postural orthostatic tachycardia syndrome (POTS) occurs in approximately 30% of people with highly symptomatic post-COVID-19 condition (PCC). It involves several symptoms that limit physical and psychological functions and cause reduced quality of life. Evidence for different treatments of POTS and PCC is limited, and this study aimed to evaluate the feasibility of individually tailored physical exercise. The secondary aim of the study was to evaluate the preliminary effectiveness of this intervention. Twenty-six participants (81% female, median age 41 years) were enrolled and performed individually tailored endurance and strength training, with progression, for twelve weeks. During the intervention period, the participants had weekly support from a physiotherapist. Feasibility was evaluated with good compliance, with 76% adherence to exercise prescription and 96% completing the study protocol. The treatment was safe, and the evaluation methods (questionnaires, physical assessments, and accelerometer monitoring) were judged to be feasible. After the intervention, improvements in symptom burden as well as in psychological and physical functions were observed. In conclusion, future randomized controlled trials can be performed with only minor adjustments and could include questionnaires, physical assessment and accelerometer monitoring, which were demonstrated as feasible by this study.

## Introduction

As of November of 2023, there were > 771 million confirmed cases of COVID-19 and almost 7 million deaths had been caused by the virus worldwide. In Sweden, the corresponding numbers were > 2.7 million cases and about 25,000 deaths^1^. There are reports of 3.7–50% of all convalescents developing post-COVID-19 condition (PCC)^2–5^. The large variation is due to different definitions, criteria and diagnosis codes used. Estimates on average suggest that about 10% of convalescents are affected^4^. The definition used for PCC from the World Health Organization will be used from here on to refer to the condition^5^. PCC can develop regardless of the severity of the initial infection^6^. Symptoms such as fatigue, dyspnea, cough, sleep disturbances, anxiety, depression and cognitive impairment are common^2,6–8^. Guidelines and recommendations for the treatment of PCC include physical exercise as well as pulmonary, cardiovascular, musculoskeletal, neurological and psychological rehabilitation interventions^9–13^. Treatment should be delivered early, using hybrid approaches and including various healthcare professionals, interventions and information depending on the individual’s needs^10^.

A condition frequently diagnosed among patients with PPC is postural orthostatic tachycardia syndrome (POTS), observed in about one-third of all affected individuals^14^. POTS is a cardiovascular autonomic dysfunction characterized by orthostatic intolerance and increased heart rate (> 30 BPM) when assuming an upright position. Common triggers for the onset of POTS can be infection, trauma, surgery, pregnancy, vaccination, or psychological stress. POTS may coexist with chronic fatigue syndrome, fibromyalgia, migraine, autoimmune diseases, anxiety, or hypermobility^15^. Common symptoms of POTS are nausea, dizziness, palpitations, fatigue, sleep disturbances, general weakness, tremors, headaches and cognitive problems^15,16^. The symptoms and comorbidities contribute to deconditioning in POTS, which leads to difficulties with activities of daily living (ADL) as well as significantly reduced health-related quality of life (HrQoL)^17–19^. Treatment recommendations for POTS include physical exercise^20–22^, increased salt and fluid intake, avoiding prolonged time in a supine position, and physical countermeasures maneuvers^23^, in addition to pharmacological treatment^24–26^. These recommendations are frequently used in clinical practice, although there is a lack of good-quality evidence for the effectiveness of the recommended treatments^27^.

POTS triggered by COVID-19 has been described as a new phenotype of POTS on which previously used treatments may not have the same effects^28–31^. Treatment recommendations mainly include therapies that have been used in non-COVID-19 POTS^29,32,33^. Despite the fairly large number of PCC patients also diagnosed with POTS, there is a lack of research on this new phenotype, and knowledge of specific interventions is even more scarce. The present study will be one of the first to address this lack of knowledge by evaluating the feasibility and preliminary effects of an individually tailored exercise program for patients with PCC and POTS. Thus, the primary aim of this study was to evaluate process feasibility. The secondary aim of the study was to evaluate the preliminary effectiveness of the intervention on outcome measures regarding physical and psychological functions, symptom burden and quality of life.

## Methods

### Trial design

A non-randomized feasibility trial was conducted to evaluate the process (i.e., recruitment, compliance, safety, and acceptability of the intervention and the measurement protocol), as well as the scientific feasibility (i.e., preliminary effects)^34,35^ of an individually tailored physical exercise program in patients with PCC concomitantly diagnosed with POTS. The initial single-subject design was changed to a non-randomized feasibility study very early in the process since the number of measurement occasions was too demanding for this group of patients. The final design followed the CONSORT guidelines for pilot and feasibility trials^36,37^. The study was approved by the Swedish Ethical Review Authority, register number 2020-02149 with amendment 2021-01657, and registered at ClinicalTrials.gov (NCT05094622). All research was performed in accordance with the Declaration of Helsinki and relevant guidelines and regulations.

### Participants

Between December 2021 and December 2022, adults (≥ 18 years) diagnosed with PCC and concomitant POTS were identified and screened for inclusion at the post-COVID outpatient clinic and the outpatient clinic for cardiology at Karolinska University Hospital, Sweden. Inclusion criteria were a PCC-POTS diagnosis and the ability to safely engage in physical exercise, as confirmed by the patient’s physician. Exclusion criteria were significant cognitive or physical impairments (compromising engagement in the intervention) and participation in any other ongoing intervention involving physical exercise at the time of recruitment.

### Procedure

Eligible participants were contacted via telephone by a physiotherapist who informed them about the study. Those interested in participating in the study were scheduled for a visit to the physiotherapy outpatient clinic at Karolinska University Hospital. At the first appointment, the participant received written and oral information about the study before signing a written informed consent. Demographic data, such as age, sex, and occurrence of previous diseases, were collected from the participants’ medical charts. Measurements of blood pressure, heart rate and saturation while at rest, during Active Standing Test (AST)^38,39^ and after a six-minute walk test (6MWT)^40^ were collected at baseline assessment. In addition to vital parameters in rest and activity, physical capacity was assessed as distance covered during the 6MWT. Questionnaires were used to assess symptom burden where fatigue was assessed with the Fatigue Severity Scale (FSS)^41^ and other disease-specific symptoms were assessed with the Vanderbilt Orthostatic Symptom Scale (VOSS)^42^ and Malmö POTS Scale (MaPS)^43^. Psychological functions such as anxiety and depression were assessed with the General Anxiety Disorder 7-Item Scale (GAD-7)^44^ and Patient Health Questionnaire (PHQ-9)^45^. Health-related quality of life was assessed by EQ-VAS^19,46^ and ability to work with the Working Ability Index^47^ (WAI). Assessments were conducted on three to four occasions, spread over two weeks at baseline, and on one or two occasions spread over two weeks after the intervention. The time frame for assessments was dimensioned according to guidelines and aimed to cover fluctuations of symptoms and unknown responses to the assessments within this new population. Each assessment session took 45–60 min, all sessions were conducted in the outpatient clinic, and all data were collected in case report forms (CRF). Measurement of participants’ habitual physical activity (i.e., steps per day) and minutes per day spent lying down, sitting, standing, and walking were measured by ActivPAL accelerometers^48,49^. Each participant wore two accelerometers, placed on the chest and thigh^50^. The participants wore accelerometers for one week in daily life (24 h/day) and used a diary to report if there were any inconveniences with the equipment or interruptions of the monitoring.

### Intervention

**Fig. 1:**
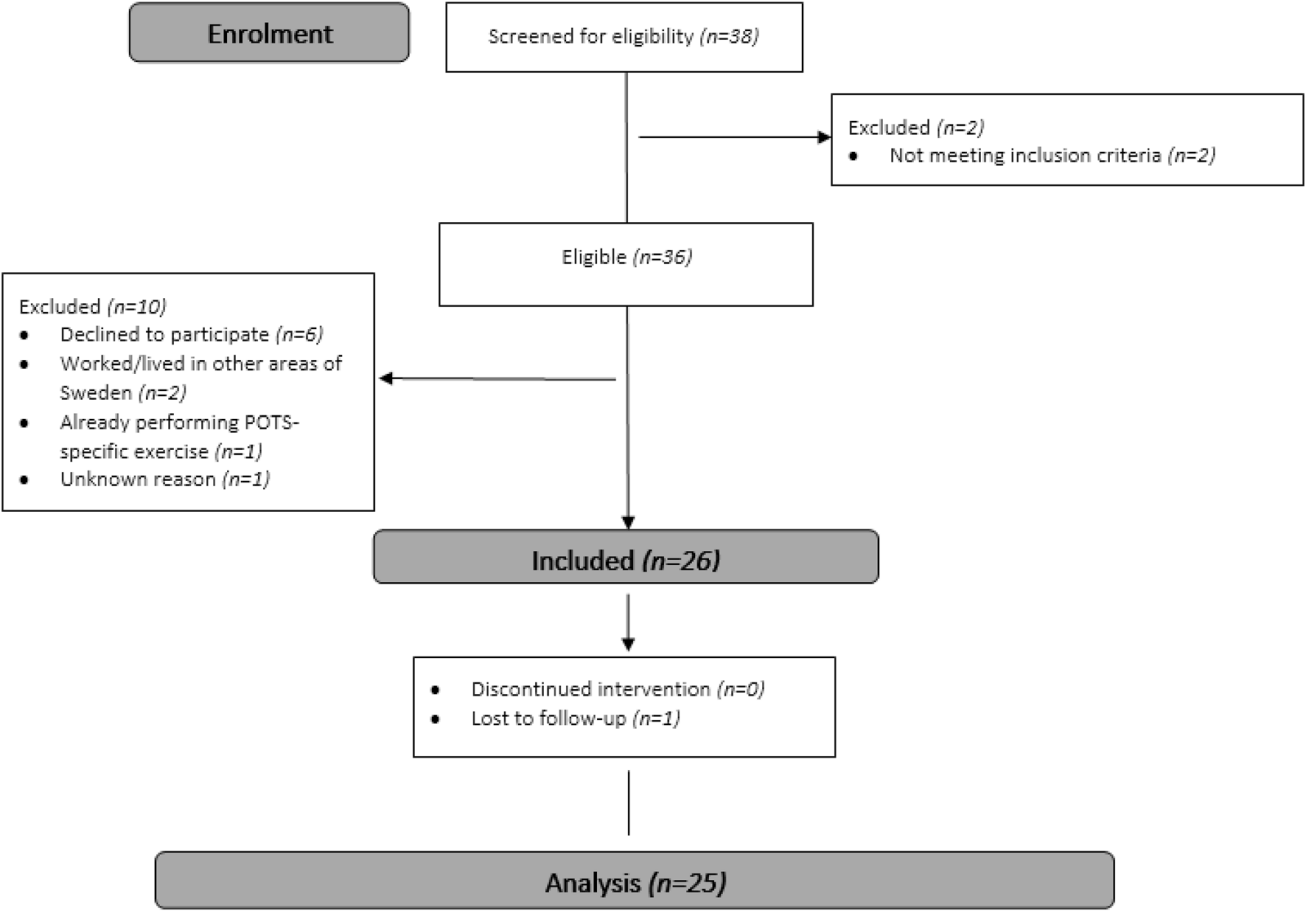
Flowchart describing the recruitment process, patient flow and compliance through the study.

The intervention consisted of endurance and strength exercises during a 12-week intervention period and was modified with respect to programs previously described by Q. Fu, S. A. George, and C. H. Gibbons^20–22^. The participants were encouraged to do endurance exercises three times a week and strengthening exercises twice a week, with no more than two consecutive days of rest. Endurance exercises aimed at light to moderate intensity (11–14 BORG rating of perceived exertion (RPE)), self-paced by the participant through the BORG RPE^51^. Endurance exercises could be performed in any posture, depending on the individual need and tolerance of the participant. However, as described in previous literature^21^ the preferred starting position was either supine or seated when these positions were tolerated by the patient. Endurance exercises gradually progressed in duration and/or more demanding posture (from supine toward upright). Individually tailored strength exercises were prescribed for each participant, aiming to improve strength in the abdomen and lower extremities, aiming for eight to ten repetitions in three sets. The dose and intensity of the strength exercises were guided by the patients' self-rated exertion, aiming for a moderate intensity (13–15 BORG RPE). An overview of the intervention and treatment are displayed in supplementary files, Fig. 1 and supplementary Table 1. The intervention included weekly contact with a physiotherapist, either at the Karolinska University Hospital or in a primary care rehabilitation facility. The intervention started with the first appointment with a physiotherapist when all baseline sessions were conducted. The physiotherapist introduced, informed and instructed each participant about the intervention with individually tailored exercise, prescribed the dose of recommended exercise, and then managed weekly contact with each participant over the following 12-week intervention period. Since the intervention was individually tailored, the setting where the intervention was performed varied between participants and could also change over time for the same participant—for example, both digital (video or telephone) and physical visits with a physiotherapist were possible depending on the participants’ needs. Most of the intervention were performed unsupervised in the participants’ homes.

At the weekly follow-up, the physiotherapist ensured adequate and safe treatment, performed structured monitoring, and reported the pace of progression, collecting feedback from the participants on the intervention and registering adverse events that occurred during the intervention period. All physiotherapists involved in the study took part in meetings before and during the intervention and received written information and a manual on how to conduct the intervention.

### Outcomes

The feasibility outcomes are summarized in Table 1.

**Table 1:** Overview of feasibility aspects.

Feasibility aspect		Data collection and outcomes
Recruitment	Screening	Number of patients assessed as eligible by physician
	Eligibility	Number of screened patients considered eligible according to inclusion/exclusion criteria
	Recruitment rate	Number of eligible patients consenting to participate
Study procedure	Adherence	Number of participants completing the intervention period and study protocol
	Acceptability of evaluation methods	Proportion of missing data for outcomes (from CRF)
Intervention delivery	Frequency of exercise sessions	Number of reported exercise sessions (from participants’ protocols)
	Frequency of physiotherapy appointments	Number of appointments (from physiotherapists’ protocols)
	Type and kind of physiotherapy appointments	Appointment specifications and/or settings (from physiotherapists’ protocols)
	Progression pace	Start and final dose of exercise reported from participants’ & physiotherapist’s protocols
	Acceptability of type and dose of strength exercise	Reported from participants' & physiotherapists’ protocols
	Patient perspectives on the intervention	Reported from participants' & physiotherapists’ protocols
Safety of procedure and intervention	Frequency of adverse events	Number reported by participants, physiotherapists and CRF
	Severity of adverse events	Reported from all protocols *Adverse event* is any unfavorable or unintended disease, sign, or symptom that is temporally associated with the intervention. An adverse event may or may not be considered related to the medical therapy or procedure^52^ *Serious adverse events (SAE)* are adverse events that result in death, a life-threatening adverse event, require hospitalization or cause a persistent or significant disability or incapacity^52^
Potential outcome measures	Primary outcomes	Physical activity and posture (ActivPAL) and health-related quality of life (EQ-VAS)
	Secondary outcomes	Physical capacity (6MWT), vital signs in rest and activity (AST, 6MWT), anxiety (GAD-7), depression (PHQ-9), fatigue (FSS), work ability (WAI)

CRF, Case Report Form; EQ-VAS, EuroQol Visual Analogue Scale; 6MWT, Six Minutes’ Walk Test; AST, Active Standing Test; GAD-7, generalized anxiety disorder 7-item scale; PHQ-9, Patient Health Questionnaire; FSS, Fatigue Severity Scale; WAI, Work Ability Index.

### Feasibility of the intervention

Safety and feasibility of the intervention were registered during the intervention period, by both participants and physiotherapists.

The physiotherapist registered weekly in a protocol what had been prescribed for each participant, the participant’s feedback and perspectives on the intervention, and information about any adverse events during the appointment or if the participants reported incidents or symptoms that had occurred during unsupervised exercise. The participants used a protocol to register compliance to prescribed exercises, perceived exertion during exercise and comments on experiences and/or any negative effects or adverse events during/caused by the intervention (Supplementary Fig. 2). Adverse events were defined according to the definition of the National Institutes of Health (NIH)^52^, where adverse events are defined as any unfavorable or unintended disease, sign, or symptom that is associated with the intervention. Serious adverse events (SAE) are defined as events that result in death, are life-threatening, require hospitalization or cause a persistent or significant disability or incapacity. Safety aspects were evaluated with the number of reported adverse events as well as with the severity of the events (from both participants’ and physiotherapists’ protocols).

The feasibility of the study procedure and intervention delivery were evaluated by the protocols—through records of performed adherence, dosage and frequency of the intervention collected from the participants’ protocols, records of the number and context of physiotherapy appointments and records of the prescriptions collected from the protocols by the physiotherapists.

### Feasibility of measurement protocol

The evaluation methods consisted of questionnaires that aimed to evaluate symptoms, quality of life and workability, as objective measurements of physical function and physical activity. The applied questionnaires were FSS^41^, GAD-7^44^, PHQ-9^45^, VOSS^42^, MaPS^43^, EQ-VAS^19,46^ and WAI^47^. The measurements of physical functions included vital signs measurement, the AST^38,39^, the 6MWT^40^, and ActivPAL^48^. Some measurements (6MWT, AST, EQ-VAS, MaPS, VOSS) were conducted twice before the intervention period to further evaluate the reliability, performance and safety of the tests.

The feasibility of safety and study procedure was evaluated by using the CRF, evaluating the number of interrupted physical tests or whether the tests could not be performed, and missing data from the questionnaires. The researchers also noted the occurrence of adverse events during and after each session of assessment.

### Analytical methods

Demographics of the participants and feasibility aspects were described as numbers, percentages, means or medians and standard deviations or IQRs, depending on the data’s measurement level. Both participants' and physiotherapists' protocols were read by two independent researchers (A.S. and A.S.R) to compile adverse events and the participants' perceptions of what affected the feasibility of the intervention. The identified adverse events were described as numbers, and factors that affected feasibility were presented to display the participants' different perceptions.

The analysis of the preliminary effectiveness of the intervention included analyses of physical function, activity, and self-reported symptoms. For the assessments that were performed on more than one occasion at baseline, an average of the two tests has been used, except for the 6MWT where the best result was used as baseline^40^. Physical activity and posture (i.e., pitch angle) data from the thigh and chest-worn ActivPAL devices were downloaded and converted to 15-s epochs using the PAL analysis software (v.8.11.8.75). The first five days with valid data were included in the analysis, except if there were reported periods of non-wearing time from the participants—such reported periods were excluded from the analyses. Average daily steps, daily stepping and time spent sedentary were extracted from the thigh-worn ActivPAL for each participant. Furthermore, the daily pitch angle data from each device was used to classify the different postures, i.e., standing (if the pitch angle for both ActivPALs was ≥ 21°), lying down- (if the pitch angle for both ActivPALs were < 21°), sitting (if the pitch angle for the thigh-worn ActivPAL was < 21° and the chest-worn ActivPAL ≥ 21°) and walking (if the pitch angles for both ActivPALs were ≥ 21° and if more than 5 steps per 15-s epoch). Subsequently, the mean time spent in various positions per day was calculated for each participant. All physical and psychological functions were analysed for differences between baseline and after the intervention, using SPSS (Version: 28.0.1.1 (14)) and paired-sample t-tests or Wilcoxon signed ranks tests depending on data measurement level. An effect or difference was considered significant if its p-value < 0.05.

## Results

**Fig. 2:**
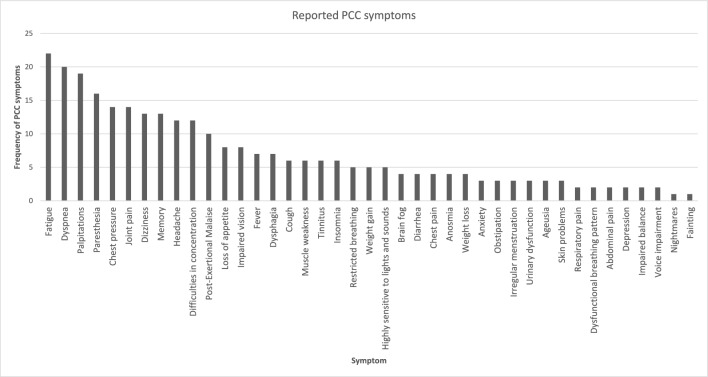
Figure showing what Post COVID-19 condition symptoms were reported by participants and their distribution, with the most frequently reported symptoms from left.

Thirty-eight potential participants were identified by cardiologists at the outpatient clinics. Two could not be included because they did not meet the inclusion criteria. Of the 36 who were screened for inclusion in the study, 26 were included, of whom all completed the intervention, but one participant did not complete the post-intervention measurement (see Fig. 1). This resulted in a 72% recruitment rate, a 100% completion rate of the intervention, and a 96% fulfilment rate for the study procedure. Baseline characteristics of participants are presented in Table 2 and Fig. 2. The majority of the participants were female (80.8%), their median age was 41 years (min–max: 21–55) and reported PCC symptoms were 13 in median (min–max: 7–26).

**Table 2:** Baseline characteristics.

Age (years); median (min–max)	41	(21–55)
BMI; median (IQR)	22.8	(7.7)
Female; % (N)	80.8	(21)
Education; n/total	Higher education	21/26
	Upper secondary school	2/26
	Missing	3/26
Occupation; n/total	Working	21/26
	Studying	3/26
	Missing	2/26
Sick leave; median (IQR)	50%	(75)
Current smoker; n/total	1/26	1/26
	Missing	2/26
Comorbidities (ICD-10); n/total	Fatigue (G933)	1/26
	Pain (S13.4, G439, M549, M791, M25.5, R529)	7/26
	Depression (F412, F329, F321)	5/26
	Asthma (J45.9, J45.P)	5/26
	Anxiety (F411, F412)	4/26
	High blood pressure (I109)	4/26
	Rheumatic disease (M073, M059L, M350B)	2/26
	Burn-out (F43.8A)	1/26
	Cancer (D136)	1/26
	Neurological disease	0
	Kidney disease	0
	Other respiratory disease	0
	Allergy	0
	Diabetes	0
	Cardiac disease	0
	Other^a^	10/26
Time since COVID-19-infection (days); mean (SD)	677	(155)
Time since POTS diagnosis (days); median, (min–max)	89.5	(9–268)
Number of PCC symptoms; median (IQR)	13	(4.5)

BMI, Body Mass Index; IQR, interquartile range, SD, standard deviation, PCC, post covid-19 condition; POTS, postural orthostatic tachycardia syndrome.

^a^IBS (n = 2 (K583)), eating disorder (n = 2 (R63.0, F50.9)), ADHD (n = 1 (F90.0B)), dysmenorrhea (n = 1, (N946)), anemia (n = 1 (D509)), lipedema (n = 1 (R60.0B)), hyperparathyroidism (n = 1, (E213)), sclerosing cholangitis (n = 1, K83.0A), urticaria (n = 1 (L509)).

### Feasibility of the intervention

**Fig. 3:**
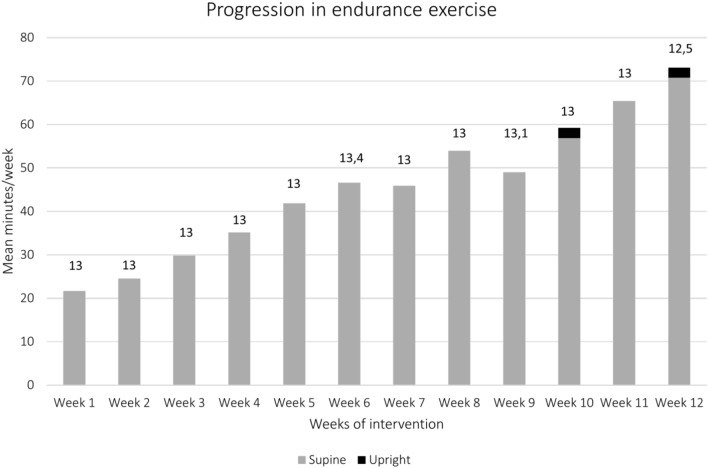
Overview of the endurance progression in mean minutes/week. Numbers above each stack indicate the median intensity, rated by BORG RPE.

Nineteen (73%) of the twenty-six participants returned their intervention protocol at the post-intervention assessment, whereof one had insufficient documentation. The median number of exercise sessions during the intervention was 51,8 (76% adherence of maximum 60 possible sessions, Min 6–Max 59), and the median progression increased from 6 to 30 min of laying endurance exercise/day (Fig. 3). The median intensity was 13 (BORG RPE) throughout the intervention period, but there was a large variation within the group (min–max: 9.6–19.0). The most common adverse event reported by the participants was headaches, which was reported a total of 45 times by seven participants, followed by different types of fatigue or malaise, reported by 2–8 participants a total of 7–24 times. No serious adverse events were reported by the participants' protocols—however, there were adverse events identified by the physiotherapists that may be considered as more severe (but did not meet the requirements for SAE), which included falls or fainting during the unsupervised exercise. The participants' feedback on the intervention is summoned in Table 3 and shows that factors in the environment and private life may affect the experiences, effects and feasibility in both positive and negative ways. The individually tailored exercise, and thereby the possibilities for adaptations, were appreciated by the participants and facilitated intervention.

**Table 3:** Summary of patient perspectives.

	Categories of perspectives	Examples
Negative experiences or effects of the intervention	Physical setbacks	Fatigue, PEM, headache, dizziness
	Less activity in everyday life	Stopped cycling to work due to fatigue
Positive experiences or effects of the intervention	Increased activity and participation in everyday life	Managing to cycle to school, being able to walk longer distances, running errands feels less strenuous
	Increased muscle strength	Increased leg strength
	Decreased dyspnea and improved breathing	Easier to take deep breaths, easier to breathe, less dyspnea
	Return to work or school	Increased attendance at school, return to work, increased working hours
	Improved mental health	Positive to exercise, feel better mentally
Factors that aggravated the intervention	Family affairs	Funeral, visiting relatives and grandchildren, travelling, legal process
	Household chores	Increased household chores when relatives being away
	Other healthcare visits, treatments and investigations	Other hospital and/or doctor visits, multiple medical investigations
	Employment	High workload, school start, ended or decreased sick leave
	Equipment, tools or environment	Broken bicycle, heat, compression tights, too many papers/protocols to keep track of the intervention
	Illness	Infections, urticaria, migraine, BPPV, PEM, stomach flu, knee surgery, sleep disturbances
	Medication adjustments	Medication adjustments
Factors that facilitated the intervention	Flexibility	Ability to change the type of training and/or exercises to everyday activities, ability to adapt training over the week to suit private life, possibility to reduce training when needed, ability to adjust the time for training (morning/afternoon/evening)
	Adaptations in endurance exercise	Inserting short breaks during exercise, reduced frequency
	Adaptations in strength exercise	Splitting exercises into two sessions to reduce muscle fatigue, avoid static exercises, fewer repetitions, perform static exercises
	Positioning	Rest and squat between exercises, ability to do exercise in supine, recumbent or sitting position
	Equipment, tools or environment	Access to a bicycle, possibilities to adapt depending on weather, access to home exercise equipment, CPAP during exercise, prescription of assistive devices, being on sick leave, compression garments
	Personal characteristics	Being motivated
	Support from the physiotherapist	Support and regular contact with the PT
	Medication adjustments	Medication adjustments

PEM, post-exertional malaise; BPPV, Benign paroxysmal positional vertigo; PT, physiotherapist.

The type and number of appointments with physiotherapists are described in the supplementary files (Supplementary material, Fig. 3). The mean number of appointments was eleven per participant, and consultation by telephone was the most frequently used type of appointment, although every participant had at least one session with the physiotherapist at the clinic. The physiotherapists prescribed a median of eight (min–max: 6–12) exercises for each participant (Supplementary material, Table 1).

### Feasibility of measurement protocol

Twenty-three of the study participants (88%) completed the measurements at baseline and post-intervention. Of those with missing data, one did not perform post-intervention assessment, one participant declined to perform the 6MWT and another participant declined to perform the AST. Of those who declined specific evaluations, both declined at the post-intervention assessment. No SAE were registered during data collection; however, participants described that POTS symptoms such as nausea, dizziness and palpitations were triggered during the AST and 6MWT and that they therefore needed to rest > 5 min before proceeding to the next assessment. The median score at VOSS after AST was 40 at baseline and 35 after intervention, indicating the severity of the symptoms during AST. During 6MWT the most described symptom was dizziness, followed by leg fatigue (Supplementary material, Table 2).

The proportion with complete data was 96–100% in the questionnaires (both pre- and post-intervention), except for the WAI questionnaire where only 68% of the participants had complete data (Table 4). The procedure feasibility showed a 100% return rate of the accelerometers, both pre- and post-intervention. There were few reported disruptions in the monitoring and non-wear time was 56.6 and 102.2 min in mean. Skin reactions such as rashes and/or itching from the dressing used for the accelerometers were reported by three of the participants. There was missing data from the accelerometers (n = 4 in total) due to technical issues caused by the devices or in the process of converting data from the accelerometers. A summary of the data quality and results from the accelerometers is shown in Table 5.

**Table 4:** Summary of missing data from questionnaires.

	1st baseline (N = 25)	2nd baseline (N = 25)	Post-session (N = 25)	% Missing of total
VOSS	0	1	2	4
FSS	0	0	0	0
MaPS	1	0	0	1
GAD	1	–	1	4
PHQ-9	1	–	1	4
EQ-VAS	0	0	0	0
WAI	5	–	9	28
PSFS	1	–	1	4

VOSS, Vanderbilt Orthostatic Symptom Scale; FSS, Fatigue Severity Scale; MaPS, Malmö POTS Scale; GAD-7, General Anxiety Disorder 7-Item Scale; PHQ-9, Patient Health Questionnaire; EQ-VAS, EuroQoL Visual Analogue Scale; WAI, Work Ability Index; PSFS, Patient Specific Functional Scale.

**Table 5:** Overview of accelerometer results.

	Pre (N = 23)		Post (N = 22)	
	Mean	SD	Mean	SD
Non-wear time, min/day	56.6	46.7	102.2	172.7
Steps/day	4891.8	3042.7	5242.1	3495.8
Stepping time	63.1	34.3	64.6	35.7
Sedentary time	1070.9	136.3	998.3	176.5

### Preliminary effectiveness of the intervention

**Fig. 4:**
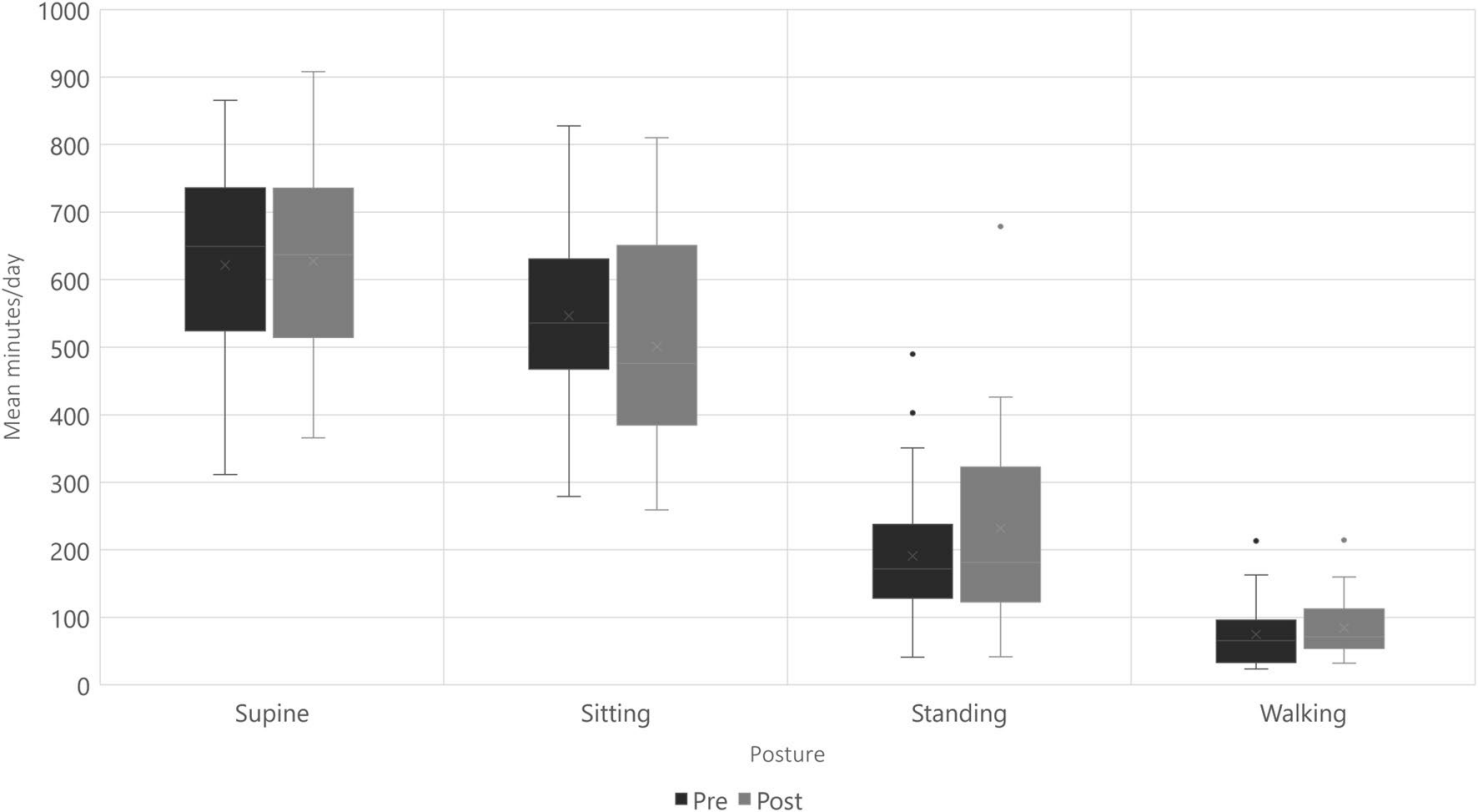
Summary of accelerometer data presented as mean minutes/day (n = 22). Figures in grey show how time in different positions was distributed at baseline and the white figures show the distribution after twelve weeks of intervention.

The 12-week intervention resulted in both improved physical and psychological functions and a reduction of diagnosis-specific symptoms. The results are displayed in Table 6. Diagnosis-specific symptoms significantly improved—for VOSS, there was an improvement from 41.4 to 32.9, FSS from 6.4 to 6.2 and for MaPS from 59.5 to 49.0 points in mean (all p < 0.05). There was no significant difference in GAD-7 (anxiety) after the intervention, but symptoms of depression (PHQ-9) had decreased from 12.5 to 9 points in median (p < 0.003). The evaluation of the participants’ physical capacity showed an improvement in walking distance (18.83 m in mean) and self-reported physical activity (from a median of 2 (IQR 1) to a median of 3 (IQR 0) at Frändin/Grimby). The objective measurements and results on time spent in different body positions are displayed in Fig. 4. The data illustrates the changes in time in various postures, which did not change significantly.

**Table 6:** Preliminary effectiveness of the intervention.

N		Pre-intervention		Post intervention		MD	95% CI		P
		Mean	± SD	Mean	± SD		Lower	Upper	
Diagnosis-specific symptoms									
VOSS (0–90)	22	41.4	17.1	32.9	19.1	− 8.47	− 15.30	− 1.65	**0.017**
FSS* (1–7)	25	6.4	0.6	6.2	1.0				**0.026**
MaPS (0–120)	24	59.5	21.7	49.0	21.7	− 10.50	− 17.78	− 3.21	**0.007**
Psychological functions									
GAD-7* (0–21)	24	4.5	10	5	6				0.137
PHQ-9* (0–27)	24	12.5	8	9	7				**0.003**
Physical functions									
HR at rest	24	71.5	13.6	75.1	16.2	3.56	0.13	6.99	**0.043**
SBP at rest	24	116.2	12.0	116.2	12.9	0.08	− 3.7	3.9	0.964
DBP at rest	24	76.0	9.1	77.4	10.8	1.37	− 1.50	4.25	0.333
SpO2 at rest	24	99.3	0.7	99.1	1.1	− 0.20	− 0.75	0.34	0.441
AST, HR at 10 min	23	92.0	15.1	93.2	20.2	1.17	− 3.96	6.31	0.641
AST, SBP at 10min	22	117.0	13.3	118.4	17.0	1.45	− 4.37	7.28	0.609
AST, DBP at 10 min	22	91.4	9.6	89.8	10.1	− 1.61	− 5.61	2.38	0.411
AST, SpO2 at 10 min	24	99.2	0.8	99.8	0.5	0.54	0.20	0.88	**0.003**
AST, RPE at 10 min*	24	15.5	4.3	14.2	4.0				0.794
6MWT, distance	24	492.8	109.1	511.7	114.9	18.83	3.61	34.0	**0.017**
6MWT, HR at 6 min	24	110.3	17.9	115.0	21.2	11.71	22.79	− 2.33	0.181
6MWT, RPE at 6 min*	24	15.5	2.2	15.0	2.0				0.542
6MWT, CR10 leg fatigue at 6 min*	19	6.0	2.5	5.0	3.0				0.810
Frändin/Grimby*	23	2.0	1.0	3.0	0.0				**0.012**
Other									
EQ-VAS	25	37.0	14.8	48.0	18.7	11.0	6.37	15.7	** < 0.001**
WAI* (7–49)	21	16.0	8	18.0	8				0.229
PSFS 1* (0–10)	24	3.0	3.0	5.0	3.0				** < 0.001**
PSFS 2* (0–10)	24	3.0	2.0	4.5	3.0				**0.001**
PSFS 3* (0–10)	11	2.0	2.0	5.0	5.0				**0.011**

MD, mean difference; SD, standard deviation; IQR, interquartile range; CI, confidence interval; VOSS, Vanderbilt Orthostatic Symptom Scale; FSS, Fatigue Severity Scale; MaPS, Malmö POTS Scale; GAD-7, General Anxiety Disorder 7-Item Scale; PHQ-9, Patient Health Questionnaire; HR, heart rate; SBP, systolic blood pressure; DBP, diastolic blood pressure; AST, Active Standing Test; 6MWT, Six Minutes’ Walk Test; EQ-VAS, EuroQoL Visual Analogue Scale; WAI, Work Ability Index; PSFS, Patient Specific Functional Scale.

*Presented as Median and IQR, analysed by non-parametric test.

## Discussion

To our knowledge, this is one of the first studies that has evaluated the feasibility of an individually tailored exercise program in patients with postural orthostatic tachycardia syndrome related to post-COVID-19 condition and regarding the study procedures. The main findings of this study were that individually tailored exercise was safe and feasible for patients with PCC-POTS. The intervention could be delivered with a satisfactory completion rate and adherence in a clinical setting. The study also provided tentative results of improved health-related quality of life and improved physical and psychological functioning over the treatment period. Thus, the interventions, protocols and outcome measures used seem feasible for a full-scale randomized controlled study.

One of the research questions was how well patients with PCC-POTS could adhere to an individually tailored exercise program in terms of intensity, training frequency and occurrence of adverse events. Overall, the tolerance for increasing position elevation and duration of exercise is considered good, since the intervention was delivered with high compliance and adherence. The participant protocols were in general adequately filled out and included documentation of the completed training, as well as patient perspectives, negative events or reasons why exercise could not be performed. Since the symptoms and functional levels of the participants varied widely, the participants' experiences show that the possibility of adapting the exercise, progression and environment most likely contributed to good adherence during the intervention period, as suggested also by previous studies^11,20^. In addition, the physiotherapists reported that it was possible to deliver the intervention in a clinical setting, including the home-based exercise, within available clinical resources and infrastructure.

The two adverse events that were considered more severe occurred during unsupervised exercise and involved falls and/or fainting, highlighting the need for a thorough risk assessment in the population, as part of the baseline assessment, to reduce risks both in future studies and in clinical treatment. Since there is uncertainty and imprecision in the probability of adverse events from previous studies in both POTS^20–22^ and PCC^11–13^, this study adds new insights and few patients reported increased symptoms, that were considered low/moderate risks or safety concerns. These symptoms are part of the disease and can thus be expected to fluctuate over time. One might speculate whether increased symptoms were caused by the intervention itself or by other possible triggers in daily life. As reported by the participants several factors in their private lives affected their possibility of carrying out the intervention. Our clinical experience with PCC-POTS patients emphasizes the importance of pacing strategies during physical exercise. This strategy has previously been used in patients with similar symptoms to PCC-POTS, for example in populations with myalgic encephalomyelitis (ME) and chronic fatigue syndrome (CFS). Pacing and slow progression have successfully been applied to reduce negative effects, post-exertional malaise (PEM), and worsening of symptoms within these populations^53–55^. For the participants in this study, a slower progression and/or lower intensity might have reduced the described negative effects further, warranting a longer period of intervention in future randomized controlled trials^56^. What appears in the participants' feedback is that activities and events in their everyday lives also affect them, hence it may be important to include support to manage their symptoms in daily life as a part of the treatment.

Symptoms that were frequently reported, during the intervention as well as at pre- and post-assessment, were mental fatigue and PEM. The only fatigue score that was part of the data collection was FSS—however, the scale did not fully seem to capture the complexity of fatigue. The results of FSS showed a tendency towards ceiling effects, which makes the instrument difficult to interpret for the population indicating that the instrument might not be sensitive enough to identify relevant changes in fatigue within this population. The study from Gibbon et al.^20^ used the Krupp Fatigue Severity Scale and reported similar difficulties with severe and persistent fatigue, and discussed whether the fatigue symptom is resistant to the graded exercise intervention, in contrast to patients with ME/CFS^54^. However, reduced fatigue was not the main objective of this study, but still seems important to assess in studies of PCC and POTS, since fatigue has a major impact on daily life^16^. Interestingly, during the post-intervention assessment and at the weekly follow-up by the physiotherapist, many of the participants reported that they experienced increased muscle strength in the lower extremities, although in this study no appropriate evaluation method for this domain was applied. An evaluation of muscle strength would be valuable to measure the effects of exercise interventions in future research.

The other questionnaires and assessments were in general considered feasible to identify important functional limitations in the studied population, and were all well accepted, since there were no interruptions or severe events during the physical assessments. The complaints of skin reactions from the accelerometer dressings are well-known side effects, and all participants were able to continue the monitoring when they received a different kind of dressing. The data quality from the accelerometers was acceptable, with few interruptions in the monitoring. This strengthens the rationale for using both questionnaires and objective measurements in future studies. Early in the process, unequivocal feedback from participants and study personnel suggested that the participants managed to perform baseline- or post-intervention tests in one session rather than spreading them out, as long there was a possibility to rest between the different tests.

The symptom burden (VOSS, MaPS, and PHQ-9) was reduced, and improvements were demonstrated in physical functions, capacity, and health-related quality of life (HR at rest, 6MWT, Frändin/Grimby and EQ-VAS) over the study period. However, these tentative findings should be interpreted with caution, since the study did not include a control group. Furthermore, it remains unclear whether these statistically significant improvements reflect an important clinical change. Previous studies in the PCC population showed a significant average improvement of 35.84 m^11^. Compared to other populations with disabilities, an increased distance of 14.0 to 30.5 in 6MWT^57^ has been judged as clinically significant, but others argue that 54 m^58^ is the minimal change to demonstrate a clinically significant improvement in functional status (e.g. among patients with chronic diseases, such as COPD). A similar caveat applies to PHQ-9 and FSS, where minimal changes of five points on the PHQ-9^59^ and of approximately 0.5–1.1 points on the FSS^60,61^ have been judged to correspond to a clinically relevant change among other populations. The study from Jaywant, A. et al. shows an average score of 9.5 (± 7.0) at PHQ-9 among the PCC population^62^ and the current cohort in this study has higher scores both before and after the completion of the intervention period. The same applies when comparing this cohort to another Swedish PCC population where the average score of FSS is 6.0^63^ and the PCC-POTS cohort scored 6.4 and 6.2 respectively. Normative data and significant improvement of HrQoL have been previously debated and adjudged minimal clinically significant differences differ between populations^64–67^, although the results from this study are comparable to the results from an Australian study^19^ that investigated HrQoL in patients with POTS.

There were no significant differences seen in posture, as measured with accelerometers. This might also be explained by the limited power of the study—or by the habits and behavior of the participants, which may take longer time to change than what the twelve-week intervention period covers. The ActivPAL provides an objective measurement of physical activity and time spent in different postures, as well as detailed information about physical activity, movement behavior, and sedentary time. Until now, the POTS population has mostly been described as deconditioned without further specifications^15,16,29,56,68^. A more in-depth description of the PCC and POTS populations’ movement patterns, activity and sedentary time would thus be valuable in future studies.

Since the preexisting knowledge of the natural course of the disease was very limited regarding individual differences, a design with a single-subject method would have been valuable, which was also the original design of this study. However, early in the process, it was noticed that the number of measurement occasions as well as the physical tests performed on each occasion were very demanding for the participants. Thus, the decision was made to change the design to a non-randomized feasibility study. The change in design meant a greatly reduced number of measurement sessions for the participants, which probably caused less fatigue and PEM compared to what a single-subject design would have entailed. Participants used self-reported protocol to document the training sessions to be able to follow compliance to treatment and this data in addition to the patient perspectives on what has facilitated the intervention provides important information for the design of future larger randomized controlled trials.

The potential for recruitment and completion of exercise training may be considered as very good, which is promising for proceeding to a full-scale randomized controlled trial (RCT)^20,21,54,69,70^. The feasibility study did not evaluate the willingness to randomization—thus there is a lack of knowledge regarding the likelihood of succeeding with enrollment to an RCT. Moreover, all participants received treatment, which might impact the estimated effect and power calculation of the primary (and secondary) outcomes for a future RCT. In this study, the participants' experiences are based on what emerged from their structured weekly follow-ups and protocols. However, to fully explore and understand patients’ perspectives and experiences of individualized physical exercise, a qualitative study is relevant to provide more enriched data on the intervention. It might be seen as a limitation that there were no predefined specified criteria for whether an instrument, procedure, or evaluation was feasible—^35,70,71^ however, analyses were made based on consensus among the researchers and results from other trials. Even though the result of recruitment rate, adherence, and compliance seem feasible compared to other studies in different populations^20–22,56,69^, the feasibility of safety and the measurement protocol could have been more robust if the limits were decided and set in advance.

## Conclusion

This study demonstrated that procedures and measurement protocol are feasible in clinical research on the PCC-POTS population. The intervention with individually tailored exercise is considered safe, feasible and acceptable. The intervention can be improved with minor adjustments for future studies to reduce fatigue, in terms of duration and progression criteria. The tentative results of this study show the effectiveness of the intervention on primary and secondary outcomes, which supports the selection of assessments in future studies. An RCT is needed to further evaluate the intervention’s effectiveness compared with the natural course of the condition. For future studies, it is also recommended that aspects of muscle function be assessed, and that fatigue should be investigated further in order to thoroughly assess interventions’ effects on physical and psychological functions.

### Supplementary Information

Supplementary Information.

## Data Availability

Data is not publicly available, but available upon request. Requests for access to the data can be put to our Research Data Office (rdo@ki.se) at Karolinska Institutet and will be handled according to the relevant legislation. This will require a data processing agreement or similar with the recipient of the data.

## Abbreviations

ADL: Activities of daily living
AST: Active Standing Test
GAD-7: General anxiety disorder 7 item
FSS: Fatigue Severity Scale
HrQoL: Health-related quality of life
MaPS: Malmö POTS Scale
PCC: Post COVID-19 condition
PEM: Post exertional malaise
PHQ-9: Patient Health Questionnaire
POTS: Postural orthostatic tachycardia syndrome
RPE: Rating of perceived exertion
SAE: Serious adverse events
VOSS: Vanderbilt Orthostatic Symptom Scale
WAI: Work Ability Index
6MWT: Six Minutes’ Walk Test
