# Supplementary material for: Individually tailored exercise in patients with postural orthostatic tachycardia syndrome related to post-COVID-19 condition – a feasibility study
Source: Sci Rep. 2024 Aug 28;14:20017. doi: 10.1038/s41598-024-71055-5 (PMC11358431; doi:10.1038/s41598-024-71055-5)
Supplement: Supplementary file 1 — Supplementary Information. [file 41598_2024_71055_MOESM1_ESM.pdf]

Supplementary material

RECOV – PCC-POTS

All sessions should include 5 – 10 minutes of warm up and cool down.

Position for endurance exercise ☐ **Supine** ☐ **Seated** ☐ **Standing/Upright**

Suitable endurance exercise: ______________________________

Week	Mon	Tue	Wed	Thu	Fri	Sat	Sun
1	3 min exercise<br>2 min rest/recover<br>3 min exercise	Strength exercises	3 min exercise<br>2 min rest/recover<br>3 min exercise	Strength exercises	3 min exercise<br>2 min rest/recover<br>3 min exercise		
2	4 min exercise<br>3 min rest/recover<br>4 min exercise	Strength exercises	4 min exercise<br>3 min rest/recover<br>4 min exercise	Strength exercises	4 min exercise<br>3 min rest/recover<br>4 min exercise		
3	5 min exercise<br>3 min rest/recover<br>5 min exercise	Strength exercises	5 min exercise<br>3 min rest/recover<br>5 min exercise	Strength exercises	5 min exercise<br>3 min rest/recover<br>5 min exercise		
4	6 min exercise<br>3 min rest/recover<br>6 min exercise	Strength exercises	7 min exercise<br>3 min rest/recover<br>5 min exercise	Strength exercises	7 min exercise<br>3 min rest/recover<br>5 min exercise		
5	6 min exercise<br>3 min rest/recover<br>6 min exercise<br>3 min rest/recover<br>6 min exercise		7 min exercise<br>3 min rest/recover<br>7 min exercise<br>3 min rest/recover<br>7 min exercise		8 min exercise<br>3 min rest/recover<br>8 min exercise<br>3 min rest/recover<br>8 min exercise		

Supplementary Figure 1 - Overview of the intervention

- Fill out your diary every day, even on days when exercise has not been performed
- Note what kind of exercises you have performed
- Use BORG RPE to rate how your perceived exertion
- Note other experiences regarding the exercises in the field for comments, t ex reasons why exercise could not be performed, illness, reactions or symptoms

Date	Activity/exercise	Duration (total minutes)	Perceived exertion (BORG RPE)	Comments

Supplementary Figure 2 - Participants protocol

Supplementary Table 1- Summary of prescribed exercises (n=23)

Exercise	The number of participants who had the exercise prescribed
Plantar and dorsal flexion of ankles	23
Lying single knee flexion	7
Elevation of pelvis in supine	23
Dead bug	9
Straight leg rises in supine	22
Clamshell (supine or side-laying)	20
Hip abduction in side-laying	14
Side plank	5
Plank	2
Plank – rolling from side to side	0
Renegade row, standing on all fours	0
Push-ups	1
Lateral hand walk standing on all fours	6
Arm lift standing on all fours	2
Fire hydrants	7
Leg lift standing on all fours	4
Leg and arm lift standing on all fours	0
Slides / double knee pulls	0
Squat toward a wall	1
Push-up toward a wall	0
Stretch – rotation of trunk	19
Stretch – lateral flexion of trunk	2
Stretch – flexion/extension of trunk	4
Stretch – protraction of chest	2
Back rising	1
Yin yoga	1
Hunter’s rest, against wall	1
Leg press	1
Contraction of buttocks and legs	2
Pilates	1

Supplementary Table 2- Summary of participant-reported symptoms during/after 6 minutes' walk test

Symptom	n 1st session	n 2nd session	n post session
Discomfort lower extremities	8	11	6
Dizziness/pre-syncope	6	12	2
Breathing difficulties	6	4	3
No reported symptoms	3	3	16
Fatigue	3	0	0
Headache	2	3	0
Weakness	2	2	0
Numbness	2	1	0
Visual disturbances	1	3	0
Chest tightness	1	2	0
Nausea	1	2	0
Fear of falling	1	0	0
Cough/hoarseness	0	1	0
Impaired balance	0	1	0

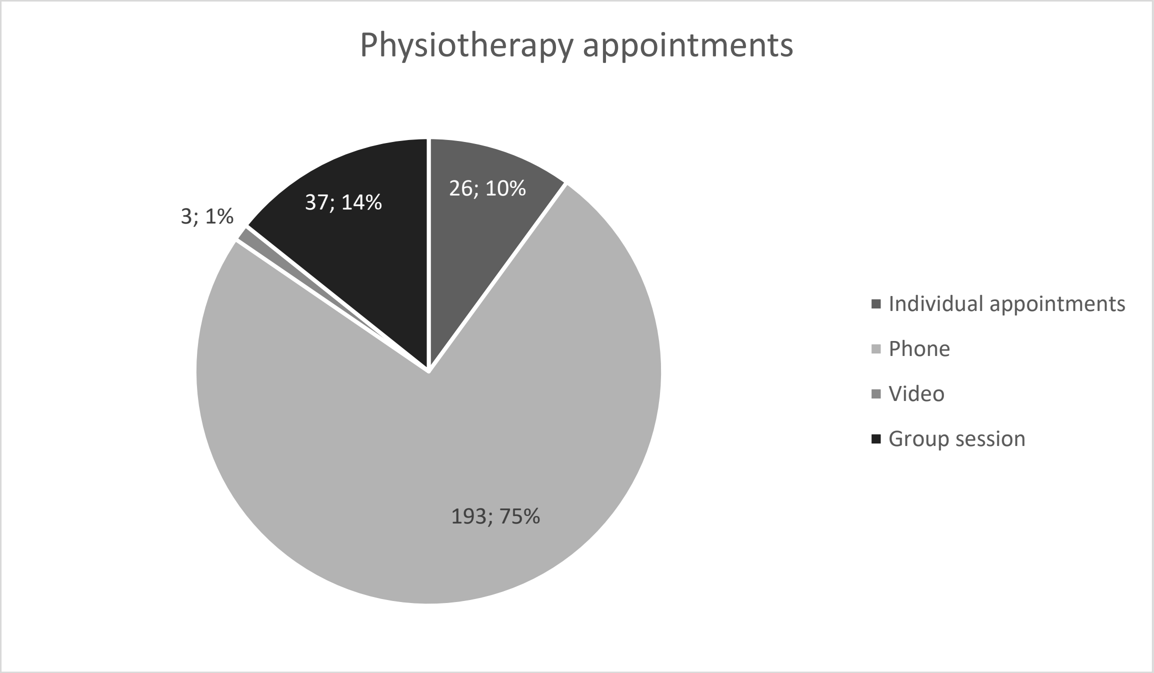

Supplementary Figure 3 - Distribution of the total numbers (n=259) of physiotherapy appointments
